# Effects of manual diaphragm release on pain, disability and diaphragm function in patients with chronic neck pain: a pilot randomized controlled trial

**DOI:** 10.1186/s12906-025-05090-8

**Published:** 2025-10-01

**Authors:** Kai-Chia Cheng, Everlynn Yi Xuan Hii, Yao-Nan Lin, Yi-Liang Kuo, Yi-Ju Tsai

**Affiliations:** 1 Institute of Allied Health Sciences, College of Medicine, National Cheng Kung University, Tainan, Taiwan; 2 PMG Healthcare Sdn Bhd, Sarikei, Sarawak, Malaysia; 3 Ru-Kang Physical Therapy Clinic, Chiayi, Taiwan; 4 Department of Physical Therapy, College of Medicine, National Cheng Kung University, Tainan, Taiwan; 5 Physical Therapy Center, National Cheng Kung University Hospital, Tainan, Taiwan

**Keywords:** Chronic neck pain, Diaphragm manual release, Ultrasonography, Fascia

## Abstract

**Background:**

Chronic neck pain (CNP) is a prevalent musculoskeletal problem associated with impaired cervical functions, faulty breathing patterns, and declined respiratory functions. Diaphragm is a critical respiratory muscle and also connects to cervical spine through different fascial connections. However, the effects of diaphragm manual release (DMR) on CNP remain unknown. Therefore, the present study aimed to investigate the effects of DMR intervention on pain, disability, and diaphragm function in people with CNP.

**Methods:**

A total of 33 participants with CNP were randomized into the DMR and sham release group (SG), and received the allocated intervention twice a week for 2 weeks. The DMR group received a firm pressure release technique at the 7th to 10th subcostal region along with deep breathing, while the SG group received the same technique with light touch instead. Primary outcomes including pain, disability, and diaphragm function, and secondary outcomes including neck range of motions, strength, and chest expansion were performed before and after the intervention for all participants.

**Results:**

After receiving 4 sessions of intervention, the DMR group demonstrated clinically significant improvements in pain and cervical range of motion, along with potential clinically improvements in diaphragm mobility. In the SG group, only pain showed a clinical significant improvement. Cervical strength and chest expansions showed potential clinical improvements in both groups.

**Conclusion:**

The current results provide preliminary evidence that the DMR shows promise as an intervention for improving pain, cervical range of motion, and potentially diaphragm mobility, cervical strength, and chest expansion in patients with CNP. However, its effects may not be clearly superior to sham intervention after 4 sessions. Future studies with larger sample sizes and longer durations are required to confirm its efficacy and establish its role in CNP management.

**Trial registration:**

The study was registered with ClinicalTrial.gov (NCT04664842) on 11/12/2020.

## Background

As the usage of electronic devices increases in modern society, chronic neck pain (CNP) has become one of the most common musculoskeletal disorders globally. The prevalence of CNP is about 37.2% from an epidemiologic systematic review in 2006 and has sharply risen to 45.7% in recent years with a higher reported rate in females [1–3]. Chronic neck pain, recognized as the fourth leading cause of disability, significantly impacts individuals causing difficulties in activities such as driving, reading, and engaging in entertainment. It leads to a poor quality of life and a high rate of work absenteeism, consequently resulting in a substantial medical burden [4–6]. Various impairments contributing to functional declines and disability have been commonly identified in patients with CNP, including reduced mobility in the cervical and thoracic spine, decreased strength and endurance of cervical muscles, weakness in deep neck flexors, and forward head posture [7–10]. In addition, patients with CNP also experience altered breathing patterns and respiratory dysfunctions. A previous study found that 83% of patients with CNP shifted their breathing patterns from abdominal to neck or upper chest [11]. Moreover, a recent systematic review including 10 studies revealed that patients with CNP had significantly poorer lung function and respiratory muscle strength compared to asymptomatic adults [12].

Several mechanisms have been advocated to explain the relationship between CNP, diaphragm, and respiratory dysfunction. Alterations in the posture and mobility of the cervical and thoracic spines may result in reduced chest expansion, and limited diaphragm excursion, potentially causing respiratory dysfunction. Overactivation of the accessory inspiratory muscles or superficial neck flexors such as sternocleidomastoid and scalenes may in turn inhibit the activations of the diaphragm. Forward head posture and/or overactive scalenes may also impede neurodynamics of the phrenic nerve and result in diaphragm dysfunction. It is also suggested that there are several fascial connections between the cervical spine and diaphragm [10, 13–15]. Any mechanical restriction in the neck myofascial system and diaphragm may adversely affect the other.

The diaphragm, a primary respiratory muscle, plays a crucial role in spinal stability and postural control [16–18]. The diaphragm contracts and descends during inspiration, and relaxes and ascends to its original shape during expiration [19]. Diaphragm mobility or excursion has been recognized as an important diaphragm function and also indicates respiration function. Diaphragm thickness or thickness change during respiration is also used to monitor diaphragm activity, detect atrophy, and indicate respiratory illness [20, 21]. Moreover, the diaphragm, together with the multifidus, transverse abdominis, and pelvic floor muscles are categorized as the deep stabilization muscles providing segmental stability for the spine. Dysfunctions of spinal stabilizer muscles can easily lead to spinal instability and result in spinal pain [22, 23]. Previous studies have found diaphragm dysfunctions in patients with low back pain [24–26]. A recent study by Hill et al. also demonstrated reduced diaphragm mobility and strength in patients with CNP compared to healthy adults [27].

Diaphragm manual release (DMR), a myofascial manual treatment aimed at reducing diaphragm tension, improving chest expansion, and facilitating the efficiency of diaphragm contraction, has been shown to benefit patients with respiratory disorders and low back pain [28–30]. Therefore, given the anatomical and neurophysiological connections between the diaphragm and the cervical spine, and the observed respiratory impairments in patients with CNP, it is hypothesized that the DMR could also benefit those with CNP.

To date, only two studies examining the effects of DMR on pain and disability in patients with CNP [31, 32]. Haghighat et al. showed that the improvements in forward head posture and chest expansion were significantly greater when adding 4 sessions of DMR to 4-week cervical exercises, but not in pain and disability [31]. In contrast, Simoni et al. did not find differences in most outcomes when receiving a combination of DMR and cervical manual therapy compared to a combination of sham release and cervical manual therapy in patients with CNP [32]. One study combined the DMR with cervical exercises, while the other combined it with other cervical manual therapy, which may produce a synergistic effect of their combined interventions. Neither study provided clear evidence of the effectiveness alone of the DMR, particularly in pain relief, an essential outcome for patients with CNP. While the available evidence is very scarce and controversial, it is currently difficult to conclude the effects of DMR intervention in patients with CNP.

Increased diaphragm mobility but not thickness following the DMR interventions have been observed in patients with chronic obstructive pulmonary disease (COPD) and healthy adults [33], but similar measures in patients with CNP are lacking. Measuring the changes in diaphragm function could provide more direct and strong evidence to support the use of DMR in patients with CNP. Therefore, the primary purpose of this study was to investigate the effects of the DMR on pain, disability, and diaphragm function in patients with CNP. In addition, the effects of the DMR on cervical range of motion, cervical strength, and chest expansion were examined.

## Materials and methods

### Study design

**Fig. 1:**
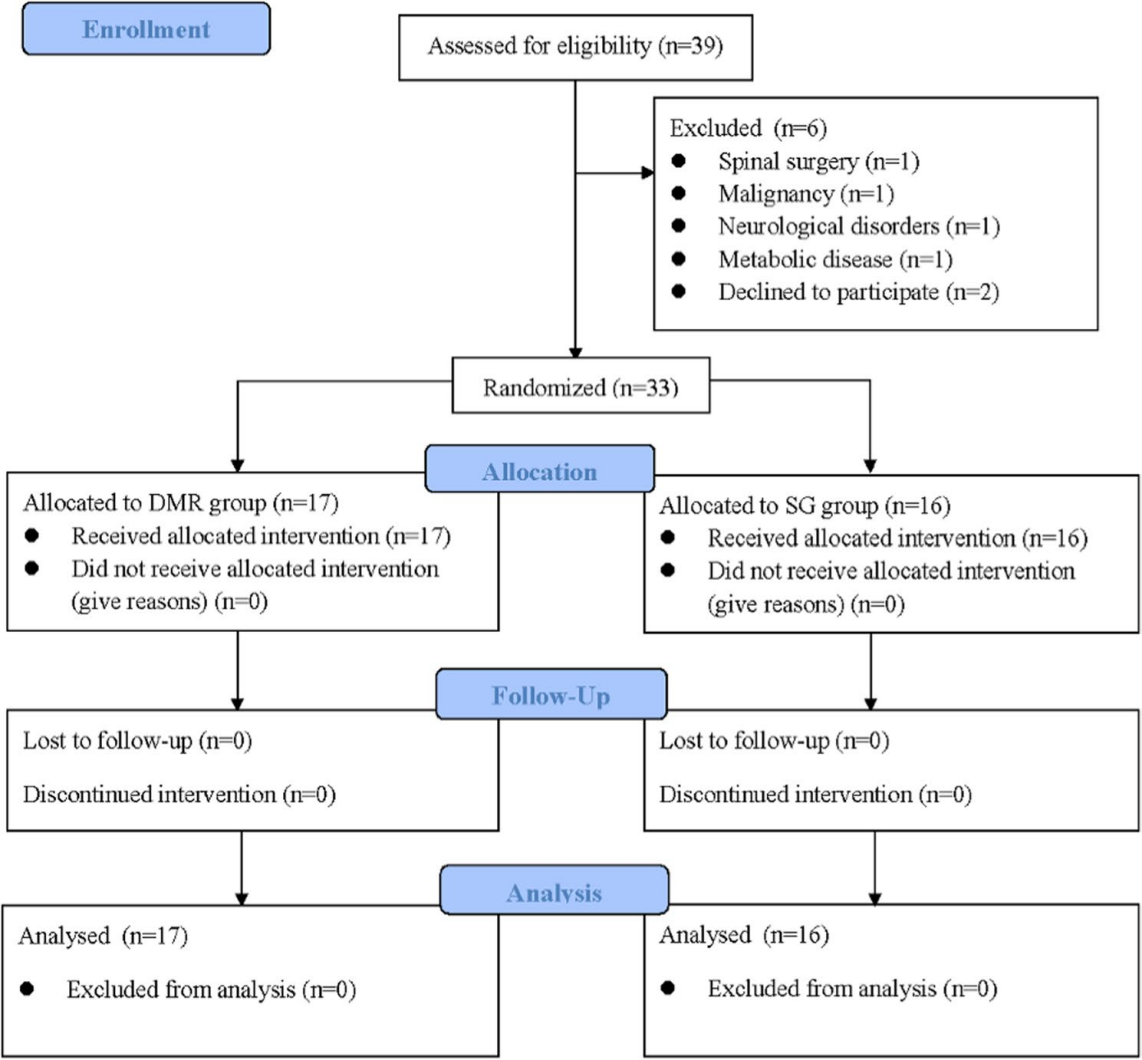
CONSORT flow diagram

This study was a double-blind, gender-stratified, and blocked randomized controlled trial (RCT). The study was conducted in Taiwan from December 2020 to December 2021. To prevent an uneven gender distribution between groups due to more females having CNP [34], participants were first stratified by gender and then centrally allocated into either the DMR group (DMR) or the sham release group (SG) using the block randomization (block size of 4) designed with Matlab software (MathWorks USA, Inc). The study was approved by the Intuitional Review Board at the National Cheng Kung University Hospital, Tainan, Taiwan (A-ER-109-159), and registered with ClinicalTrial.gov (NCT04664842) on 11/12/2020. The study design and reporting followed the guidelines outlined in the Consolidated Standards of Reporting Trials (CONSORT) 2010 statements (Fig. 1) [35]. In addition, it is important to know that this study was conducted during the period of COVID-19 pandemic, all personnel including the assessor, therapists, and participants were requested to wear masks and gloves correctly and maintain social distancing as possible during the experiment to protect personal respiratory hygiene. Comprehensive disinfection was also carried out after each experimental session.

### Participants

Participants aged between 20 and 60 years with CNP were recruited from local clinics, medical centers, and communities. To be eligible for inclusion, individuals had to experience neck pain for at least 3 months within the past year, pain localized between the upper nuchal line and T1 spinous process with or without radiating sensation and rated with a pain intensity higher than 3 based on the Numerating Rating Scale. Chronic neck pain could have resulted from specific cervical disorders, idiopathic development, or traumatic consequences. Exclusion criteria included the first onset of acute neck pain, deformities such as torticollis, scoliosis, barrel chest, pectus excavatum, and severe kyphosis, and previous surgeries in the spine and shoulder regions, malignancy, cardiopulmonary diseases, immune and metabolic disorders, neurological disorders, psychiatric diseases, smoking, pregnancy, and participation in spinal stabilization exercises such as exercises specifically targeting local stabilization muscles such as transverse abdominis and multifidus, sling exercises, gyrokinetic exercises, and Pilates within the last 12 months. To prevent the interference of poor-quality sonographic images, participants who have a body mass index (BMI) over 30 were also excluded [36]. Furthermore, this study was performed during the COVID-19 pandemic, participants who were suspected, currently, or previously infected with COVID-19 regardless of the symptom severity were all excluded. Before participating, all participants signed the approved informed consent.

The sample size was determined following the recommendations for pilot studies [37]. A recommended sample size is 10 participants per treatment arm. Accounting for a 20% dropout rate, a minimum of 12 participants per group was required.

### Interventions

**Fig. 2:**
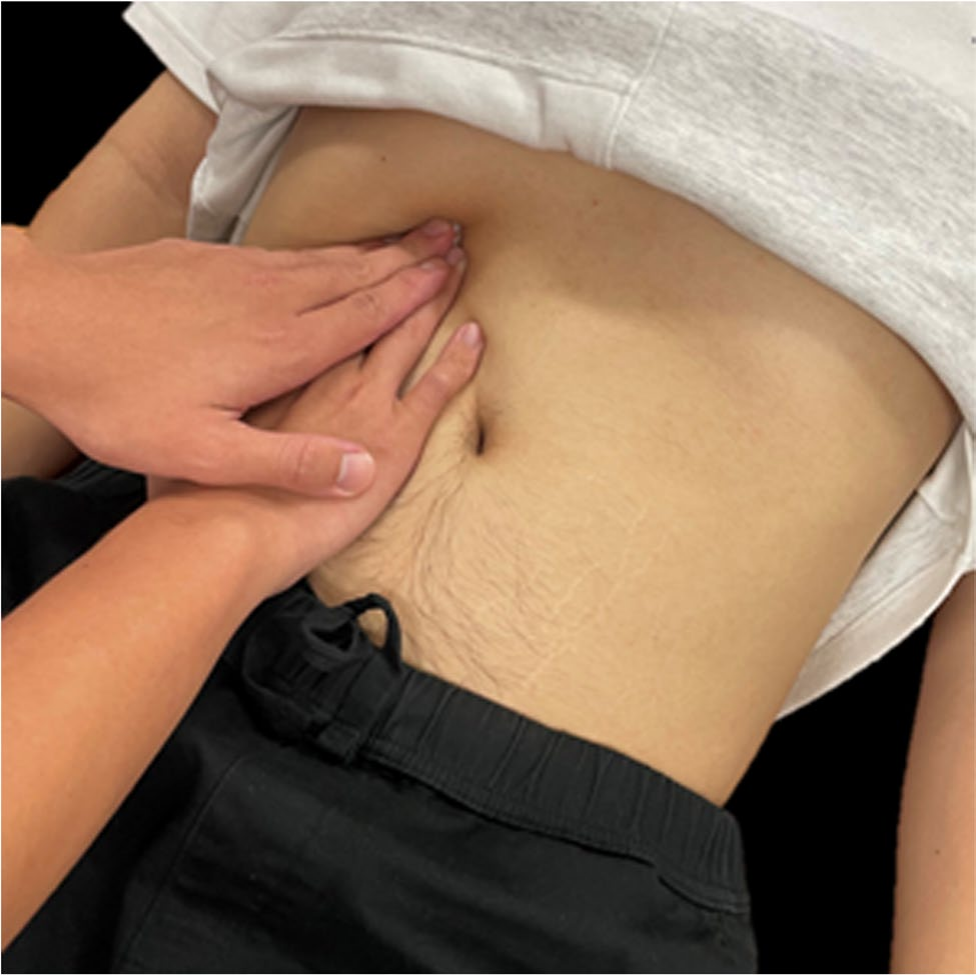
Diaphragm manual release in right 7th to 10th subcostal region

All participants received the intervention twice a week for 2 weeks by two experienced physical therapists who have been trained and dedicated in providing manual therapy for more than a decade. Before the experiment started, therapists were trained and ensured to be able to apply a relatively constant force for one minute by pressing a force platform with their fingers, simulating the techniques used in this study. Participants in the DMR group underwent the DMR intervention. Participants were requested to be crook lying with both hips and knees bent on the plinth. The therapist positioned themselves on the side of the participant and mainly used their fingers to firmly contact one side of the subcostal region between the 7th and 10th rib (Fig. 2). While the participant exhaled, the therapist applied firm pressure into the inner space of the costal margin to stretch the diaphragm. The therapist maintained this force and slowly reached into a deeper space during the whole period of expiration [28]. Following, while the participant started to inhale, the hands of the therapist stayed in the same location against the resistance from the descending diaphragm. Ten continuous breaths per set for three sets were performed for one side of the diaphragm, and interventions were administrated bilaterally. One-minute resting interval between each set was allowed. Participants in the SG group received sham release interventions with the same location, technique, and dosage as the DMR group; however, the therapist only provided a light touch at the same targeted location without applying any deeper pressure or resistance during the whole intervention period.

### Outcome measures

All participants received the same outcome measurement before and after the intervention, including both the primary outcomes (pain, disability, and diaphragm function), and secondary outcomes (cervical range of motion, cervical strength, and chest expansion). All outcome assessment was performed by the same therapist who has 3 years of clinical experience and 2 years of ultrasonography training. The assessor was not involved in the intervention and did not know the group allocation.

#### Primary outcomes

##### Pain and disability

Pain intensity was assessed using the Visual Analog Scale (VAS). Participants were instructed to mark their current pain intensity in a 100-millimeter horizontal line with no pain at 0 mm and the most severe pain labeled on the opposite side. The longer distance from the scale of no pain indicates a more severe pain. The VAS has been proven to be a reliable and valid tool for assessing pain [38, 39]. The minimal clinical importance difference (MCID) for pain as evaluated by VAS has been reported to exceed at least 6 mm in patients with neck pain [40].

Disability level was assessed using the Neck Disability Index (NDI), which is a 10-item self-reported questionnaire. Each item contains 6 descriptions for different disabled levels and is scored from 0 to 5. The total score of NDI was often converted into 100%. The higher score of the NDI indicates a higher level of disability. The reliability of NDI is good, with an intraclass correlation coefficient (ICC) of 0.87 and moderately correlated with the SF-36 [41]. The MCID for disability evaluated by NDI was 10.5% [42].

##### Diaphragm function

Diaphragm function in this study was indicated as the thickness and mobility of the diaphragm and measured using ultrasonography (ACUSON NX3TM, Siemens Solution, USA, Inc). Diaphragm thickness was captured under the B mode using a 4–12 MHz linear transducer, and diaphragm mobility was recorded under the M mode using a 1–5 MHz curved transducer. Participants were positioned in a 30° semi-recumbent supine position with 90° knee bending, and the right side of the diaphragm was measured [43, 44]. Five images were recorded for each outcome or condition and then measured using the ImageJ software. The intra-rater reliability for the current study has been examined in a pilot study on 10 young adults and is good to excellent (ICC_3,3_ = 0.804 to 0.986).

**Fig. 3:**
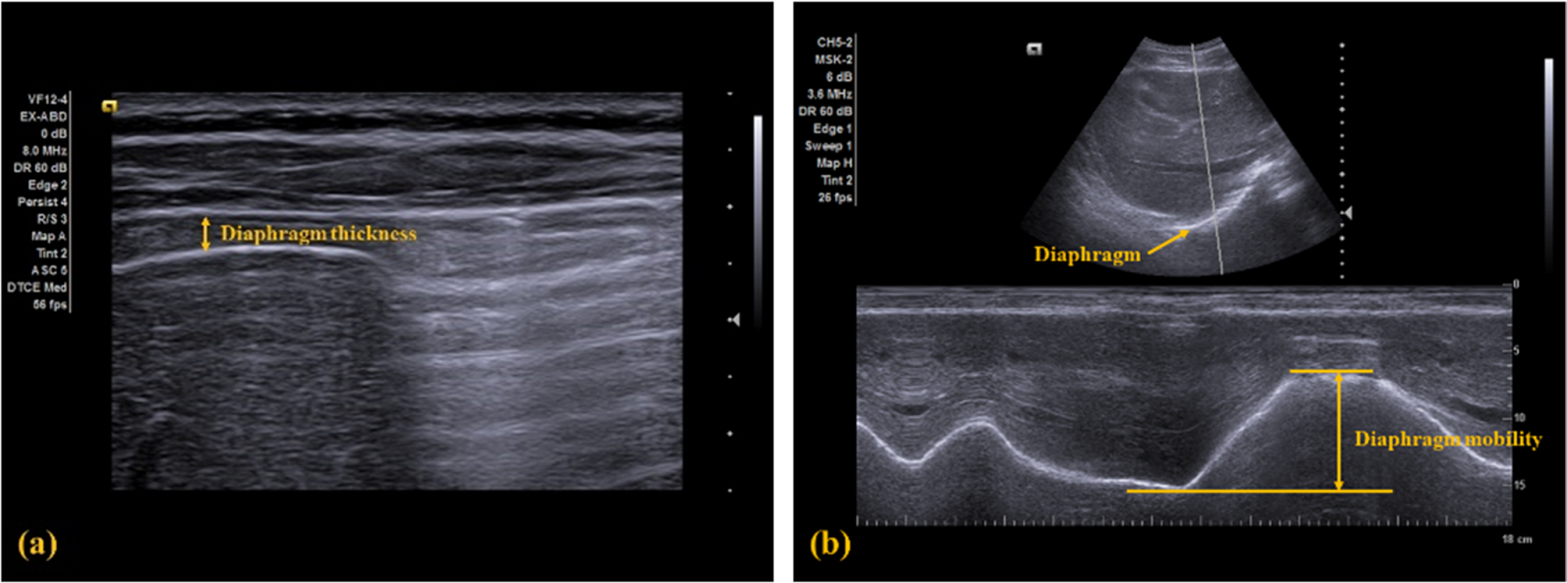
**a** illustrates the diaphragm thickness during maximal inhalation, represented by the double arrow line; **b** shows the diaphragm mobility in the lower part of the figure, indicated by the double arrow line

For the diaphragm thickness, the linear transducer was placed between the anterior and medial axillary line at the diaphragm apposition zone, parallel with the rib bones. The images of diaphragm thickness were taken at the end of the maximal inhalation and exhalation (Fig. 3a**).** Diaphragm thickness was measured as the hypoechoic distance between the hyperechoic pleural and peritoneal lines in millimeters. The diaphragm thickness change between the maximal inspiration and expiration was then calculated. The MCID for the diaphragm thickness measured via ultrasonography in patients with CNP has not yet been established.

For the diaphragm mobility, the curved transducer was placed at the anterior subcostal region on the mid-clavicular line, with the craniocaudal central line passing through the gall bladder. A hyperechoic line indicating the diaphragm was visualized in the ultrasonogram (Fig. 3b). For measuring the mobility, participants were instructed to perform two tidal breaths, followed by a maximal inhalation and exhalation. Diaphragm mobility was defined as the displacement between the highest (i.e., maximal inhalation) and lowest (i.e., maximal exhalation) positions of the diaphragm and recorded in millimeters. The MCID for the diaphragm also has not been established.

#### Secondary outcomes

##### Cervical range of motion and strength

Cervical range of motion was assessed using the Cervical Range of Motion (CROM) device (Performance Attainment Associates, Lindstrom, MN, USA). The reliability of this CROM device for measuring the cervical active range of motions has been proven to be good to excellent in previous studies (ICC = 0.89 to 0.98) and highly correlated with electromagnetic 3-D motion analysis [45]. Participants sat upright on a firm chair with their knees flexed at 90 degrees on the ground and without any back support. To standardize the testing position, participants were requested to cross their hands in front of the chest. Participants were then requested to actively perform maximal flexion, extension, bilateral side flexion, and bilateral rotation for the cervical range of motion. Three trials were performed and recorded in degrees in each direction and averaged for analysis. The MCID values for the CROM in patients with CNP were 6 and 4 degrees for cervical flexion and extension, 3 and 5 degrees for cervical right and left side flexion, and 10 and 5 degrees for cervical right and left rotation, respectively [46]. Cervical strength was assessed using the hand-held dynamometer (Micro FET3, Hoggan Health Industries, Inc, NV, USA). The validity and reliability of this hand-held dynamometer for measuring isometric strength have been proven to be adequate to excellent in previous studies [47]. Participants assumed the same seated position as during range of motion testing [48]. Maximal isometric strength in kilogram during the 3-second contraction for cervical flexion, extension, and bilateral side flexion was recorded. Three trials were performed in each direction and averaged for analysis. To prevent fatigue, participants were allowed to rest for 1 min between each trial and 5 min between each direction of movement. The MCID for cervical strength measured using a hand-held dynamometer in patients with CNP has not been established.

##### Chest expansion

Chest expansion of the upper, middle, and lower chest during the maximal inspiration and expiration was assessed using a ruler tape in centimeters, which is a common and acceptable clinical measurement for chest mobility. The reliability of the tape measurement for chest expansion has been proven to be good to excellent in previous studies (ICC = 0.89 to 0.92). This measurement was also shown to have moderate to strong correlations with lung function (*r* = 0.40 to 0.78) [49]. The measuring locations for the upper, middle, and lower chest were the third intercostal space, the xiphoid process, and the midpoint between the xiphoid process and the umbilicus, respectively. Three measurements were taken for each region and averaged for analysis. The MCID for chest expansion in patients with CNP also has not been established.

### Statistical analysis

All data were presented as Mean and standard deviation (SD) except for the gender distribution. Given the underpowered nature of the pilot study, which made it challenging to obtain significant differences, descriptive analysis was conducted using mean difference, 75%, 85%, and 95% confidence intervals (CIs), along with effect size to report the results [50]. Effect size was calculated and presented using Hedge’s g for each outcome measure, as it is sensitive to small sample sizes. To evaluate the treatment effect of DMR, the mean difference was compared to both the null effect and reported MCID. For outcomes without a reported MCID, the comparison was made using an effect size of 0.40 [51]. Clinical importance was defined when the mean difference and the effect size exceeded the reported MCID or 0.40, and the CIs did not include the null effect, while potential clinical importance was considered when the CIs included the null effect. In contrast, possible statistical changes that were equivocal to clinical importance was identified when the mean difference and the effect size did not exceed the MCID or 0.40, but the CIs did not include the null effect. Finally, no clinical meaning was defined when the mean difference and the effect size were smaller than the reported MCID or 0.40, and the CIs included the null effect.

## Results

A total of 39 patients with CNP were initially enrolled, 6 participants were excluded, and 33 participants finished and included in the final analysis (Fig. 1). No participants who plan to enroll or in the progress of the study were excluded due to the infection and suspected infection with the COVID-19. The demographic data and subject characteristics between the two groups are presented in Table 1. Most participants in this study were young to middle-aged females. The majority were graduate or undergraduate students and office workers who spent long hours in sitting or using electronic devices each day. Approximately 60% of participants in both groups regularly exercised at least once a week.

**Table 1:** Demographics and subject characteristics between the two groups

	DMR (*n* = 17)	SG (*n* = 16)
Gender (Males/Females) ^a^	4/13	3/13
Age (years)	40.12 ± 10.35	39.75 ± 10.57
Height (cm)	163.65 ± 6.85	162.69 ± 4.56
Weight (kg)	61.53 ± 9.86	61.28 ± 8.74
BMI (kg/m^2^)	22.85 ± 2.35	23.12 ± 2.92
Regularly exercise (yes/no)	11/6	10/6
Sitting time (hours/day)	8.26 ± 3.79	9.00 ± 3.32
Computer usage time (hours/day)	4.68 ± 4.42	6.09 ± 3.69
Mobile phone usage time (hours/day)	4.44 ± 2.55	4.06 ± 2.89
Pain frequency (days/week)	4.76 ± 2.17	4.25 ± 2.46

**Fig. 4:**
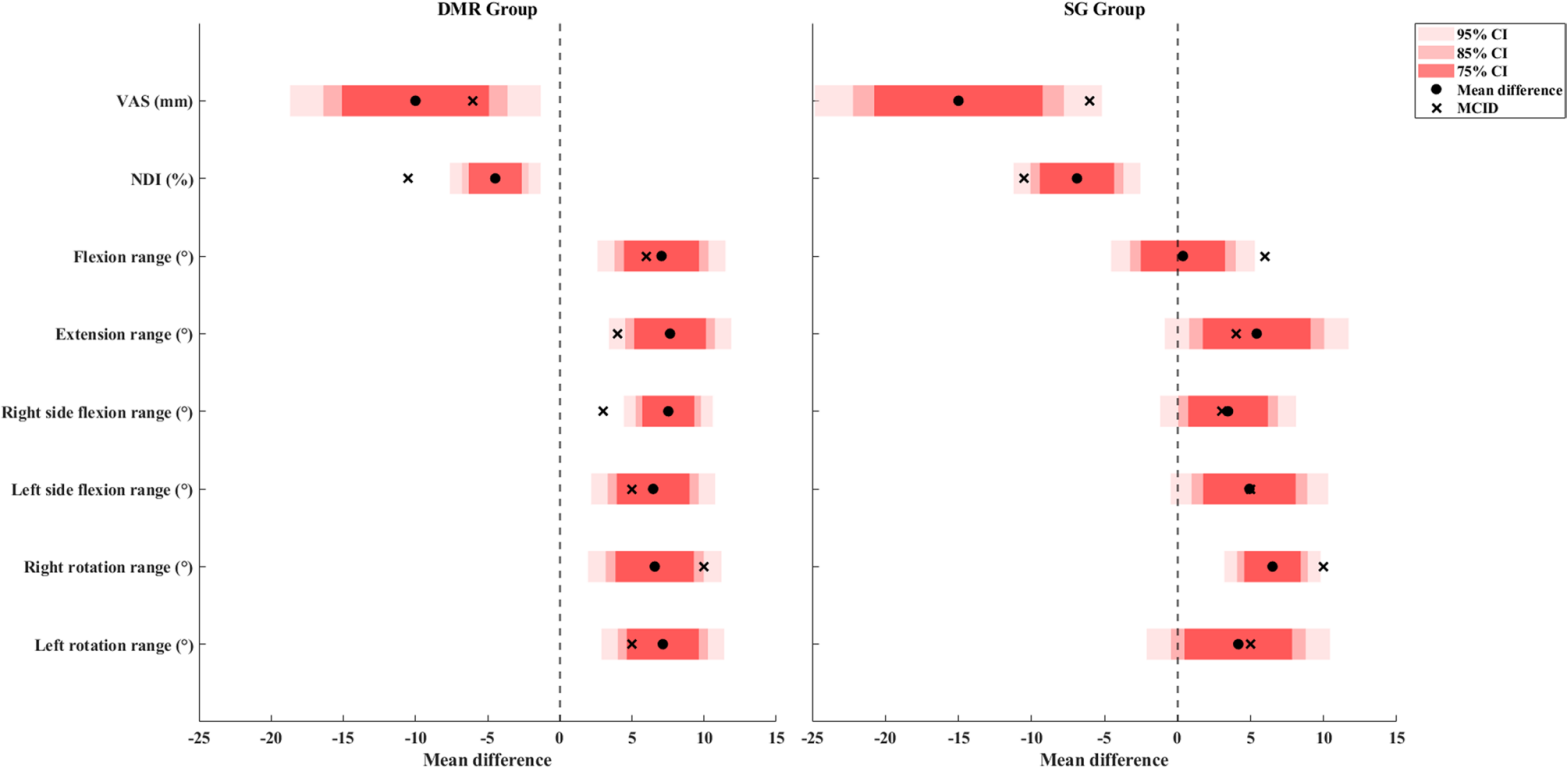
Treatment effects for outcomes in comparison to the reported MCID and null effect (black botted line)

**Fig. 5:**
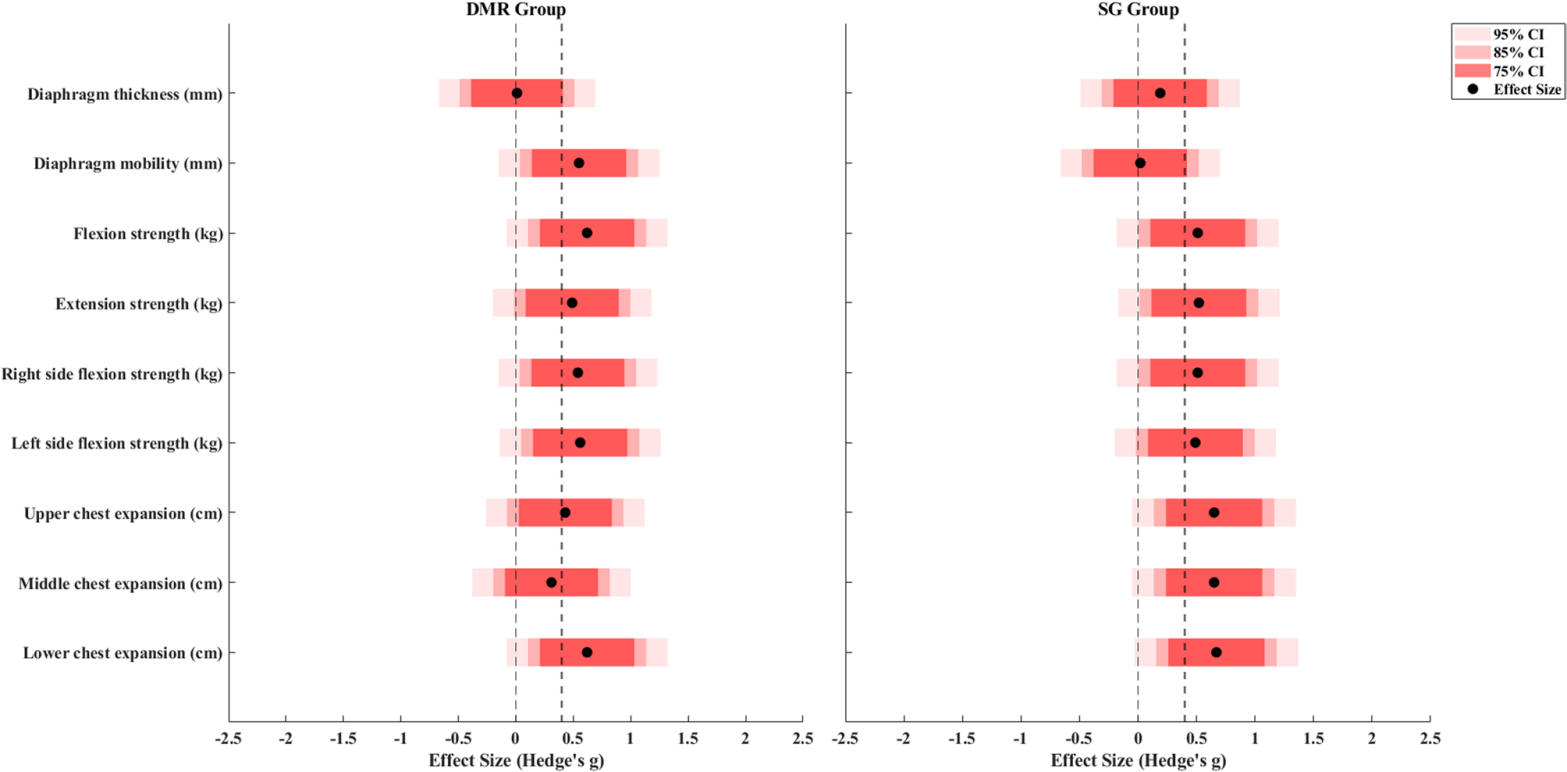
Treatment effects for the outcomes in comparison to the effect size and null effect (black dotted line)

Table 2 and Table 3 reports the descriptive results for the primary and secondary outcomes, respectively. Figure 4 illustrates the treatment effects in comparison to the reported MCID and null effect, while Fig. 5 compares the treatment effects to the effect size of 0.4 and the null effect. The mean difference in VAS exceeded the reported MCID (g = 0.49 for the DMR group and 0.68 for the SG group), and the CI did not include the null effect, indicating clinical importance. The mean difference in NDI did not exceed the reported MCID (g = 0.53 for the DMR group and 0.67 for the SG group), and the CI did not include the null effect, indicating possible statistical improvement but equivocal to have clinical importance. The change in diaphragm thickness showed a small effect in both groups (g = 0.01 for the DMR group and 0.19 for the SG group) that did not exceed 0.4, and both the CIs included the null effect, indicating no clinical meaning. The effect of diaphragm mobility in the DMR group exceeded 0.4 (g = 0.55), and the CI included the null effect, suggesting potential clinical importance. In contrast in the SG group, the effect did not exceed 0.4 (g = 0.02) and the CI also included the null effect, indicating no clinical meaning.

For the DMR group, the mean difference in flexion, extension, bilateral side flexion, and left rotation exceeded the MCID (g = 0.48, 0.76, 0.99, 0.75, and 0.71, respectively) and did not include the null effect, indicating clinical importance. For the SG group, the mean difference in extension and right lateral flexion exceeded the MCID (g = 0.32 and 0.33, respectively) but included the null effect, indicating potential clinical importance **(**Fig. 4**)**. The effects for all other secondary outcomes including strength and chest expansion in both groups were slightly larger than 0.4 but included the null effect, indicating potential clinical importance. Although middle chest expansion in the DMR group showed no clinical meaning.

**Table 2:** Descriptive results for primary outcomes

	DMR group (*n* = 17)				SG group (*n* = 16)			
	Pre	Post	MD (95% CI)	ES ^a^	Pre	Post	MD (95% CI)	ES ^a^
Pain and disability								
VAS (mm)	29.88 (19.97)	19.88 (19.81)	−10.00 (−18.69, −1.31)	0.49	38.62 (21.24)	23.63 (21.57)	−14.99 (−24.81, −5.18)	0.68
NDI (%)	16.47 (9.04)	12.00 (7.52)	−4.47 (−7.61, −1.33)	0.53	19.50 (10.31)	12.62 (9.49)	−6.88 (−11.21, −2.54)	0.67
Diaphragm function								
Thickness (mm)	1.87 (1.48)	1.88 (1.26)	0.01 (−0.45, 0.42)	0.01	1.16 (0.50)	1.26 (0.54)	0.1 (−0.35, 0.15)	0.19
Mobility (mm)	75.00 (14.67)	85.12 (21.15)	10.12 (−17.32, −2.90)	0.55	72.50 (12.01)	72.74 (13.83)	−0.06 (−6.13, 5.64)	0.02

*Pre* Pre-intervention, *Post* Post-intervention, *MD* Mean difference, *CI* Confidence interval, *ES* Effect size represented by Hedge’s g

^a^ Within group difference

**Table 3:** Descriptive results for secondary outcomes

	DMR group (*n* = 17)				SG group (*n* = 16)			
	Pre	Post	MD (95% CI)	ES ^a^	Pre	Post	MD (95% CI)	ES ^a^
Cervical range of motion (degrees)								
Flexion	56.19 (9.26)	63.25 (7.39)	7.05 (2.63, 11.48)	0.48	52.04 (11.21)	52.41 (11.82)	0.37 (−4.53, 5.28)	0.03
Extension	65.88 (10.02)	73.52 (9.56)	7.64 (3.41, 11.87)	0.76	68.37 (16.33)	73.79 (16.05)	5.42 (−0.88, 11.72)	0.32
Right side flexion	39.84 (6.55)	47.37 (8.20)	7.53 (4.46, 10.59)	0.99	40.20 (10.02)	43.66 (10.20)	3.46 (−1.20, 8.11)	0.33
Left side flexion	44.15 (8.71)	50.62 (8.15)	6.47 (2.16, 10.77)	0.75	40.37 (12.79)	45.29 (11.49)	4.92 (−1.78, 10.31)	0.39
Right rotation	68.98 (6.84)	75.56 (8.28)	6.58 (1.95, 11.21)	0.85	67.45 (11.84)	73.95 (13.55)	6.5 (3.20, 9.79)	0.93
Left rotation	69.56 (9.92)	76.70 (9.69)	7.14 (2.87, 11.40)	0.71	69.79 (10.90)	73.95 (16.27)	4.16 (−0.10, 10.43)	0.81
Cervical strength (kg)								
Flexion	11.81 (4.14)	14.52 (4.27)	2.71 (0.79, 4.61)	0.62	11.10 (3.37)	12.96 (3.76)	1.86 (0.61, 3.11)	0.51
Extension	14.42 (4.74)	16.73 (4.41)	2.31 (0.51, 4.12)	0.49	12.99 (3.59)	15.30 (4.96)	2.31 (0.13, 4.47)	0.52
Right side flexion	12.33 (4.34)	14.54 (3.72)	2.21 (0.64, 3.77)	0.54	10.86 (2.88)	12.50 (3.38)	1.64 (0.15, 3.12)	0.51
Left side flexion	12.04 (4.03)	14.34 (4.08)	2.30 (1.15, 3.42)	0.56	10.92 (2.74)	12.48 (3.45)	1.56 (0.09, 3.03)	0.49
Chest expansion (cm)								
Upper chest	5.77 (1.48)	6.38 (1.28)	0.61 (0.22, 0.99)	0.43	5.58 (1.61)	6.72 (1.78)	1.14 (0.62, 1.65)	0.65
Middle chest	5.98 (2.62)	6.88 (2.99)	0.9 (0.23, 1.56)	0.31	5.44 (1.44)	6.58 (1.94)	1.14 (0.37, 1.89)	0.65
Lower chest	5.20 (2.42)	6.62 (2.02)	1.42 (0.41, 2.42)	0.62	4.84 (2.26)	6.25 (2.34)	1.41 (0.67, 2.13)	0.67

*Pre* Pre-intervention, *Post* Post-intervention, *MD* Mean difference, *CI* Confidence interval, *ES* Effect size represented by Hedge’s g

^a^ Within group difference

## Discussion

This study was the first double-blind pilot RCT investigating the effects of DMR on pain, disability, and ultrasonographic measures of diaphragm function in patients with CNP. The DMR intervention resulted in clinically important changes in both pain intensity and cervical range of motion. It also demonstrated potential clinical improvement in diaphragm mobility and most secondary outcomes including strength and chest expansion, highlighting its overall effectiveness. In comparison, the SG intervention showed clinically important changes in pain but did not achieve similar improvements in cervical range of motion, with only several motions indicating potential clinical improvement. Although strength and chest expansion in the SG group also showed potential clinical improvement. Overall, the DMR group exhibited more consistent and meaningful improvements compared to the SG group. The current results provide preliminary evidence that the DMR might have positive effects in patients with CNP on reducing pain and increasing diaphragm mobility, cervical range of motion, cervical voluntary isometric strength, and chest expansion. Further investigation with a larger sample size and a refined study design are warranted.

The current study found that the DMR intervention demonstrated clinically important improvements in pain and equivocal improvements in disability, although, the sham release also showed similar improvements in pain and disability. The findings suggest that the DMR intervention may have a positive effect on clinical symptoms. However, they also indicate that even light touch from the therapist, combined with cycles of deep breathing performed by the participants during the sham release intervention could produce therapeutic effects. These effects might result from placebo effects associated with manual contact and parasympathetic relaxation induced by deep breathing [52, 53]. It is possible that 4 sessions of the DMR intervention or the dosage or duration of each session in this study might not be enough to induce more significant improvements than the sham release intervention, especially if deep breathing exercises were involved. Breathing with tactile stimulations may induce more neurophysiological effects to promote relaxation and reduce pain, primarily through activating the parasympathetic nervous system and inhibiting the sympathetic nervous system. These effects are typically immediate due to direct nervous system stimulation but are often temporary, as they do not result in lasting structural changes [54]. A longer duration or more sessions for the DMR intervention to induce permanent structural adaptation for the diaphragm and cervical spine might be suggested for future studies to provide more solid evidence for the application of the DMR in patients with CNP.

The current study is the first to demonstrate the effects of applying DMR on diaphragm function in patients with CNP. The findings revealed that diaphragm mobility measured by ultrasonography tended to be greater in the DMR group compared to the SG group. Notably, a potentially clinical significant improvement in diaphragm mobility was observed only in the DMR group, but not in the SG group. Previous studies in patients with CNP did not measure diaphragm function after receiving the DMR intervention [31, 32]. It remains uncertain whether the improvements in forward head posture and chest expansion observed in the study of Haghighat et al. could be attributed to the improved diaphragm function resulting from the DMR techniques [31]. The current results support the hypothesis of fascial connections between the diaphragm and cervical spine, and align with previous studies measuring diaphragm mobility in healthy adults and patients with COPD [28, 33, 55]. Mancini et al. showed a significant increase in diaphragm mobility in healthy adults after receiving a single session of the DMR compared to the sham and control interventions, with no changes in diaphragm thickness [55]. Studies suggest that the application of the DMR may directly induce biomechanical changes in the diaphragm [17, 30], in addition to the neurological effects of active breathing. No changes in diaphragm thickness were expected in this study, as the DMR intervention did not focus on muscle strengthening or breathing training that could lead to activation or morphological changes. While the findings in this study align with previous research, significant differences between the DMR and SG groups remain limited, highlighting the need for larger studies with refined methodologies to confirm these results and further clarify the potential benefits of the DMR on diaphragm function in CNP.

In addition to increased diaphragm mobility, the DMR group demonstrated improvements in most cervical range of motions with clinical important changes. In contrast, the SG group showed only potential improvements in a few motions without similar clinical meaningful changes. These findings suggest that the DMR intervention may have positive effects on improving cervical range of motions. To date, only two previous studies have investigated the effects of the DMR in patients with CNP; one did not measure the cervical range of motion [31], and the other found no changes in the cervical range of motion [32], making direct comparisons difficult. The potential mechanisms by which DMR could improve cervical range of motion are multifaceted and interrelated, including releasing tension in the fascial systems surrounding the cervical and thoracic regions, facilitating diaphragmatic breathing, reducing the workload on cervical muscles, providing overall spinal stability, activating the parasympathetic nervous system, and reducing pain [13, 56–60]. Studies have indicated the diaphragm interacts with the cervical spine through several fascial connections including the thoracolumbar fascia, transversalis fascia, and deep and median cervical fascia. The thoracolumbar fascia originates from the lumbosacral region, extends through the thoracic region, and firmly attached to the cervical region. In the cervical region, it covers the cervical paraspinal muscles, merges with the ambient fascia, and eventually attaches to the cranial base [56]. The transversalis fascia originates from the median and deep cervical fascia, descends to the pubis, and firmly attaches to the transversus abdominis muscle, which derives as a continuation of endothoracic fascia and thus is considered to connect with the diaphragm and contributes to the stability of the entire spine [13]. It is believed that any tightness or shortening of the structure along these fascial lines could lead to restrictions or impairments in other structures including the diaphragm [13, 57, 58, 61]. Additionally, diaphragm movement is believed to be correlated with and contributes partially to chest expansion, which is also a part of the fascial line [62]. Some studies have found that applying manual therapy on one structure along the fascial line resulted in an increased range of motion or mobility of the remote structure along the fascial line [59, 60]. Marizeiro et al. found that 2 sessions of the DMR intervention significantly improved chest expansion, respiratory muscle strength, flexibility of posterior chain muscles, and lumbar range of motion more than a placebo intervention with only light touch in sedentary healthy young women [60].

Although the clinical changes in cervical strength and chest expansion showed similar in both the DMR and SG groups, there are several mechanisms to explain the improvements observed after the DMR intervention are also provided. The diaphragm is innervated by the phrenic nerve, which originates from the C3-C5 nerves at the cervical spine. The application of the DMR stimulates the mechanical receptors of the diaphragm, results in the depolarization of sensory afferent neurons, and then sends the sensory information of mechanical pressure or touch to the dorsal horn. According to the gate control theory, neck pain may be inhibited since the middle neurons in the dorsal horn can be suppressed when the mechanical messages are transmitted from the diaphragm. This mechanism may partly explain the pain-relieving effects observed with the DMR [63, 64]. Moreover, the application of the DMR may also help to normalize the length-tension relationship of the diaphragm, which thereby may improve the contraction ability of the diaphragm and directly enhance the stability of the lumbar spine [17, 30]. The spine is a kinetic chain including lumbar, thoracic, and cervical regions for performing human postures and motions, and each region is interdependent [32, 65]. An increase in diaphragm function may directly provide a stronger foundation to the cervical spine and consequently lead to improved cervical functions such as greater cervical motions and strength observed in the present study.

Several limitations in the present study need to be acknowledged. The order of outcome assessment in this study did not randomize which may result in fatigue or learning effects for the participants. Future studies could randomize the sequence of the testing procedures to minimize this potential bias. A total of 4 sessions of the DMR intervention in two weeks may not be enough to induce more improvements than the sham release intervention if deep breathing is involved. A control group performing deep breathing only, or a control group receiving DMR without deep breathing, could potentially diminish the potential neurophysiological effects and provide more robust evidence of the DMR. Other control comparisons such as education, electrotherapy, and similar techniques without cues of deep breathing could also be the other alternative. In addition, future studies could include follow-up analysis to evaluate the immediate and long-term effects of the DMR intervention. The DMR technique applied in this study differed from those in previous studies. The techniques and dosages varied across research, making it unclear which technique or dosage is superior. This could be determined in future studies. Furthermore, future studies with improved design and a larger sample size are important to provide stronger evidence for investigating the effects of DMR in patients with CNP. Confounding factors such as marital status, income, occupation, and physical activity level contributing to a higher prevalence and incidence of CNP have not been comprehensively controlled in this study [66]. Future research should aim to thoroughly evaluate these factors to eliminate any potential bias. The current study focused on patients with CNP regardless of their causes of neck pain. Participants in this study were also of a wider age range and with relatively mild pain and disability. The effects of DMR in patients with diverse causes or diagnoses, specific age ranges, or those experiencing higher levels of pain and disability merit further investigation. Finally, the current study was conducted during the COVID-19 pandemic, potentially introducing influences on the participants such as psychological stress, physical inactivity, and participation bias. The effects of DMR in individuals previously infected, especially in those with severe respiratory symptoms remain uncertain. The current findings should be interpreted with increased caution.

## Conclusion

Many clinicians administrate DMR techniques in their practice to facilitate breathing, relieve fascial tension, and reduce pain despite a lack of scientific evidence to support these practices. The current study demonstrated clinical improvements in pain and cervical range of motion, as well as potential improvements in diaphragm mobility, cervical strength, and chest expansion, after receiving a 2-week DMR intervention in patients with CNP. In contrast, the sham release showed clinical improvements in pain and potential improvements in some cervical range of motion but lacked broader effects. This pilot study contributes to the emerging evidence on the role of the diaphragm in CNP management. Despite the lack of a clear superiority of DMR over sham release in some outcomes suggests the need for caution in interpreting these results. Further research with a larger sample size, a longer intervention duration, a modified study design to isolate the effects of DMR more distinctly from those of deep breathing exercises, or a multimodal program for multifactorial causes for CNP is still necessary to conclusively ascertain the efficacy of DMR in patients with CNP.

## Data Availability

The data that support the findings of this study are available from the corresponding author upon reasonable request.

## Abbreviations

CNP: Chronic neck pain
DMR: Diaphragm manual release
SG: Sham group
VAS: Visual analog scale
NDI: Neck disability index
ICC: Intraclass correlation coefficient
CROM: Cervical range of motion
SD: Standard deviation
Pre: Pre-intervention
Post: Post-intervention
CI: Confidence interval
